# Promoting Sustainable Wellbeing: Integrating Positive Psychology and Environmental Sustainability in Education

**DOI:** 10.3390/ijerph17196968

**Published:** 2020-09-23

**Authors:** Tammie Ronen, Dorit Kerret

**Affiliations:** 1 Department of Social Work, Faculty of Social Sciences, Tel-Aviv University, Tel Aviv-Yafo 6997801, Israel; Tamie@tauex.tau.ac.il; 2 Department of Public Policy, Faculty of Social Sciences, Tel-Aviv University, Tel Aviv-Yafo 6997801, Israel

**Keywords:** positive psychology, sustainability, environmental education, wellbeing literacy, character strength, hope, positive education

## Abstract

This article proposes an integrative policy approach to defining and promoting wellbeing through the joint lenses of positive psychology and environmental sustainability. The study suggests that while both positive education and environmental education address various aspects of wellbeing, a common definition is still absent. The study proposes a framework for advancing a mutual concept of wellbeing: “sustainable wellbeing”, integrating aspects of individual wellbeing and the wellbeing of the environment. Sustainable wellbeing is achieved when improving individual wellbeing is correlated with improving the wellbeing of other members of society and the natural environment. It suggests a framework for integrating the benefits of positive education and environmental education into a coherent approach for exploring, discussing, and experiencing sustainable wellbeing. The paper mainly develops, explores, and demonstrates ten rules for implementing sustainable wellbeing literacy in schools, based on cognitive behavioral therapy and positive psychology insights. It contributes to the development and understanding of wellbeing, highlights the benefits of parallel developments of two distinct educational fields, and offers practical guidelines for implementing educational programs. Furthermore, the paper contributes to developing 21st century educational systems and further develops the emerging field of positive sustainability.

## 1. Introduction

What do we want for our planet and its inhabitants? This timeless philosophical and ethical question has been at the core of modern public policy, at all levels, for many decades [1]. While improving “wellbeing” would be a common response to this query, the meaning of this highly used term remains vague and debatable [2]. Policymakers in various arenas have endeavored to define this term and find indicators to measure it [3]. The search for alternatives to the conventional 20th-century utilization of gross domestic product (GDP) as a major indicator of wellbeing focusing on economic growth led to the realization that the quality of human life is intertwined with the quality of the environment [4,5,6]. This realization resulted in numerous international and national initiatives such as the United Nations’ “sustainable development goals,” the Organization for Economic Cooperation and Development’s “better life index,” and the New Economic Foundation’s “happy planet index” [4,7,8,9].

Consequently, several studies and theoretical frameworks conceptualized and studied the connections between positive psychology and sustainability as the main disciplines underlying the various concepts of wellbeing [10]. While some studies explored the connections at the policy level [5], other conceptualizations addressed the psychological level [6,11,12,13,14], and some focused on the educational level [15,16]. Particularly, the connection has been addressed through the lens of nature connectedness [17,18,19,20,21,22,23,24].

But what is wellbeing? And how can it be achieved? In this paper, we propose an integrative policy approach to defining and promoting both human and environmental wellbeing, through the joint lenses of positive psychology and environmental sustainability. First, we outline the main differences and commonalities in the definitions of wellbeing, from the perspectives of positive psychology and of sustainability. Then, we describe the role of the educational system in promoting wellbeing, while focusing on the approaches upheld by positive psychology education and by environmental education. Our paper ends by proposing a holistic sustainable wellbeing model that integrates positive psychology and environmental sustainability using cognitive-behavioral therapy principles and offering a new language of sustainable wellbeing literacy [25] to approach thoughts, emotions, and behavior. This much-needed joint model may offer cost-effective means for 21st-century education systems to simultaneously enhance students’ personal wellbeing while promoting responsible global citizens to care for the planet’s future.

## 2. Wellbeing

Many scholars have observed that the term “wellbeing” varies in meaning between and within disciplines such as psychology, sustainability, and health domains [11,26]. Sustainability science and positive psychology can be perceived as twin conceptual approaches in that they both share the same goal of promoting wellbeing [1,9]. However, the meaning of wellbeing is not necessarily identical in the two disciplines.

In positive psychology, wellbeing is among the many concepts employed regarding the human ability to conduct a full, rich life, such as happiness, satisfaction with life, finding meaning, and flourishing [26,27]. Lindsay presents a thorough investigation of the various concepts and definitions of wellbeing, and its relationship with positive psychology [26]. While various approaches focus on different aspects of wellbeing, we will embrace, for the sake of this paper, the more holistic concept of wellbeing, integrating all of the above. Diener, Oishi et al. defined wellbeing as a combination of cognitive and emotional aspects “experienced by people according to their own subjective evaluations of their lives. These evaluations include cognitive judgments about life satisfaction, and affective reactions to life” [28] (p. 153). Accordingly, subjective wellbeing often encompasses a set of assessments that measure life satisfaction, positive affect, happiness, and low negative affect [29]. Keyes and Ryff [27] and Keyes [30,31] claimed that attaining high levels of wellbeing enables people to flourish in both personal and environmental (social) ways, including components like developing self-acceptance, finding purpose and meaning in life, experiencing continuous personal growth and mastery, and establishing positive, meaningful relations with others and with the environment.

Hence, the positive psychology perspective focuses squarely on increasing the individual’s wellbeing within the context of the environment in which that individual lives. O’Brien claimed that this focus of psychology on the individual might lead to some cases where increasing an individual’s wellbeing could come at the expense of other people or at a cost to the natural environment [16].

Environmental sustainability offers a variety of definitions for wellbeing that adopt a more systemic rather than individual approach, incorporating views of time, society, and the biosphere [1,2]. However, wellbeing in sustainability is also a highly vague term. For example, the Brundtland Report of the World Commission on Environment and Development specified that the current generation’s wellbeing should not harm future generations’ wellbeing, while specifically focusing on the term “needs” [32]. “Wellbeing is always related to the fulfilment of needs, and to say that something is needed implies an end that is considered good.” [1] (p. 23). Still, the interpretation of how “needs” relate to wellbeing remains unclear and has been the subject of much debate [2]. For instance, should wellbeing include only the minimum needs that satisfy human wellbeing? Or should it enable humans to exploit as much as possible without infringing future generations’ ability to do the same? Particularly the meaning of wellbeing was criticized for its extensive focus on economic wellbeing while neglecting the linkages between the natural environment and human flourishing [1].

As pursuing wellbeing is a core component of both positive psychology and environmental sustainability, each of these disciplines can benefit from the other’s advantages in defining and operationalizing wellbeing [2]. For example, the sustainability framework can benefit from the precise measurement tools used to assess wellbeing in positive psychology, whereas positive psychology can benefit from the contextual systems thinking inherent to sustainability science [2]. Moreover, a holistic integration of the sustainability perspective into definitions of individual wellbeing may also answer the call to use a systems approach in positive psychology [9,33]. Insights from both disciplines could help better define wellbeing in a way that includes the interdependencies occurring between people and natural systems [1,2,34].

**Figure 1:**
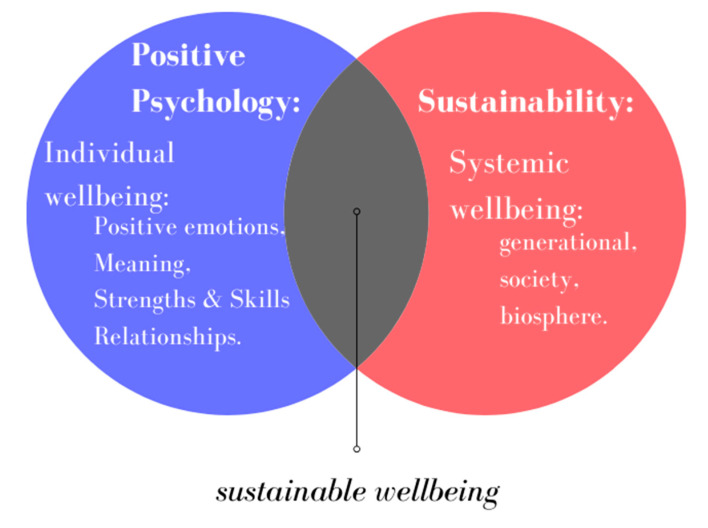
Sustainable wellbeing components.

Importantly, the establishment of a mutually acceptable or interdisciplinary definition of wellbeing holds implications for its successful implementation. We propose that a holistic definition of wellbeing using a “relational” approach may be optimal, focusing on improving individual wellbeing while considering interactions with other members of society and with the natural environment [1]. Sustainable wellbeing is achieved when improving individual wellbeing is correlated with improving the wellbeing of other members of society and the natural environment. This holistic definition, which we term “sustainable wellbeing”, is compatible both with complex systems thinking [33] and with positive psychology and environmental sustainability (See Figure 1). Following this approach, human needs, societal needs, and environmental needs are considered interrelated and interactive. This definition reflects the notion that wellbeing is not merely personal, as every individual is embedded in social structures and communities and faces multiple social tasks and challenges [30]. In addition, the “sustainable wellbeing” approach coincides with the World Health Organization’s [35,36] definition of mental health as a state of emotional wellbeing in which individuals not only identify their personal capacities, manage to cope with normative life stresses, and work efficiently and productively but can also contribute to their communities and to their natural environment.

At the operational level, a good starting point to promote sustainable wellbeing would be to implement the approach while targeting children and adolescents through the schools’ education system. School students can easily learn and accept new concepts and habits. They will, also, become the next generation in charge of their own self-care and of influencing the wellbeing of the world we live in. Furthermore, schools are prominent in shaping children’s attitudes and behaviors and thereby influencing the entire society. Therefore, schools play a major role both in defining wellbeing goals and in achieving them [9]. The following sections elaborate first on positive education as the main venue for promoting subjective wellbeing and then on environmental education as influencing the relationship between the individual and the natural environment (as summarized in Table 1). Finally, we propose a practical approach for implementing a common language for promoting sustainable wellbeing (as summarized in Table 2).

## 3. Positive Education

Researchers’ focus on wellbeing and the rise of positive psychology has emphasized personal and environmental resources and skills [27,37]. Such positive psychology approaches have translated into the educational system (often referred to as “positive education”, or “positive schooling”) [38]. While positive psychology is a way to relate to life in general, positive education implements components of positive psychology in schools. Schools have been shown as effective not only in fostering the expected cognitive development of their students but also their social and emotional growth [39,40,41]. As a place where children spend many hours of their day with their peers, schools not only impart knowledge but also serve as a living lab for social and emotional behavior. Therefore, schools serve as an essential tool for teaching and promoting wellbeing. Within the school setting, children experience opportunities for positive peer interactions, significant relationships with adults other than their parents/caregivers, and the promotion of social, emotional, and environmental learning [41,42].

Many schools now offer well-developed programs aimed at building “positive education,” which are implemented across the board by teachers [43]. Some of the programs include both intervention and prevention components. Studies have underscored the importance of positive education programs focusing on relationships, self-control, social support, meaning, and positive affect to help children and adolescents cope with difficulties and become more resilient [44,45]. Therefore, most projects oriented toward positive education aim to impart skills for facilitating wellbeing. These programs may target teachers and students. For example, a program aiming to foster teachers’ interpersonal relationships with students and parents focused on skills for enhancing teachers’ strengths (e.g., curiosity, creativity) and sense of meaning [46,47]. A comprehensive meta-analysis of positive schooling conducted recently by Waters and [48] identified six pathways of intervention (SEARCH) that effectively increase students’ wellbeing and school-based academic outcomes: Identified strength, Emotional management, Attention and awareness, Relationships, Coping, and Habits and goals.

To be noted, aimed at advancing students’ wellbeing through interventions, positive education has traditionally focused on the individual. Nevertheless, schools can enable children to experience, implement, and practice skills that will enable them not only to attain personal wellbeing but also to become caring environmental citizens. The following section briefly describes how environmental education addresses the concept of wellbeing while highlighting the differences between positive education and environmental education.

## 4. Environmental and Sustainability Education

Growing awareness of anthropogenic activity’s hazardous impact on the natural environment, and increasing knowledge about the limits of the earth’s biospheric system have led to the recognition of environmental education’s importance [49]. Environmental education has been a focus of the educational system since the late 1960s and was declared a global goal in the Tbilisi Declaration of 1977 [50]. Initially, environmental education’s objective was to foster concern and commitment to solving environmental problems [49,51]. The focus was on the wellbeing of the natural environment (the ecosystem or biosphere) and not on individuals’ wellbeing [49,52]. Nevertheless, the many strands of environmental education at least hold an implicit assumption that humans’ wellbeing is interconnected with the wellbeing of the natural environment [51]. Furthermore, many studies link various concepts of connecting with nature and individual wellbeing [17,18,20,53,54,55], particularly within the educational setting [56,57,58,59,60].

Hence, it could be claimed that both environmental education and positive education hold the same goal of increasing the wellbeing of the individual, the society, and the natural environment but that their starting points are opposite. Positive education assumes that promoting an individual’s wellbeing will increase overall social good, whereas environmental education assumes that promoting the natural environment’s wellbeing will increase the individual’s wellbeing. One of the main insights of environmental education literature is the importance of promoting nature connectedness that is tightly related to environmental behavior [22,23,24]. Promoting nature connectedness through the educational system is an important component of improving sustainable wellbeing as it is correlated both with individuals’ wellbeing and with pro-environmental behavior.

To be noted, in the field of environmental education, the terminology has generally not revolved around wellbeing, but rather, the stated goal of environmental education has typically been to foster students’ environmental “literacy.” Literacy is a relatively new term developed initially for reading and writing and then adopted by other fields to denote fluency or mastery in the subject [61]. In the environmental field, particularly in environmental education, the notion of “environmental literacy” has been widely explored since its inception in the late 1960s [61]. Promoting environmental literacy is a consensual main goal of environmental education, which has been declared in United Nations commitments [61,62]. As McBride et al. [61] (p. 3) noted, a widely accepted definition of environmental literacy is that of the North American Association for Environmental Education (2004): “an awareness of and concern about the environment and its associated problems, as well as the knowledge, skills, and motivations to work toward solutions of current problems and the prevention of new ones.” [61]. Still, the interdependence between human wellbeing and environmental quality is one of the main conventions of environmental education and is, therefore, an inherent concept of environmental literacy [63].

Environmental literature explored in-depth pathways to increase environmental literacy. Despite many insights regarding effective ways to increase environmental literacy, much is still unknown and under debate [9,64]. McBride systematic review (of environmental literacy, eco-literacy, and ecological literacy) highlighted four essential components of environmental literacy, in addition to knowledge: affect (an emotional component), cognitive skills (that include planning), environmentally responsible behavior, and self-control or internal locus of control [61]. In research on environmental education, much attention has been given to learning or teaching methods. For instance, a recent extensive review of climate change education revealed that two program components are particularly effective: providing personally relevant and meaningful information for learners and engaging the learners [65]. At the same time, although the importance of skills and competencies in building environmental literacy is widely acknowledged [49,66], fewer empirical studies have been conducted to determine which skills affect environmental literacy and how interventions may promote those skills. In this regard, environmental education could well be inspired by positive education that has focused more on improving specific identified skills and competencies (such as emotional management, using character strengths, developing habits, achieving goals, etc.) and directly measuring them [48].

Finally, a main strand of environmental education has focused on the “whole school” approach, generating international and national certification systems [67]. From this perspective, the terms green schools, eco-schools, enviro-schools, and even sustainable schools are used interchangeably to reflect an approach where the curriculum, the school management, and the community are involved in providing students with environmental education [68]. For example, as of 2019, the UNESCO international certification program recognized 59,000 schools in 68 countries as “eco-schools” [69]. While these institutional setting numbers, and the world-wide participation rates are highly impressive, research calls for a more thorough investigation of these schools’ program contents to better explain differences in their performance [67].

## 5. Positive Sustainability Education: An Integrative Approach

Environmental education and positive education have a mutual goal in common: to promote wellbeing. Each of the two fields contains some within-discipline focus alongside the call to learn from additional disciplines [48,64]. Although each field has developed significantly along separate paths over previous decades, initial attempts to integrate positive and environmental education efforts have emerged [9,15,16,70,71,72,73].

Different schools currently implement various programs to implement some of the theoretical principles of positive education [74] or environmental education [68]. Some leading schools even implement programs imparting both of these types of knowledge, sometimes separately without any connections between the programs, and at other times embracing the holistic concept of a “living school” that inherently integrates both sets of concepts while focusing holistically on humans’ wellbeing [16,75]. However, the prevailing international certification system for the whole school approach to sustainability has not yet incorporated program goals that integrate positive education principles into environmental education [67].

The current article focuses on connecting insights from both positive and environmental education perspectives to propose an integrative approach to enhancing sustainable wellbeing (see Table 1). To this end, we propose using the benefits of each field, as elaborated in Table 1. The integrated approach (“positive sustainability”) adopts a joint definition of “wellbeing” (sustainable wellbeing), focusing both on the natural environment and the individual while striving to create sustainable wellbeing literacy.

Wellbeing literacy is defined as “the vocabulary, knowledge and skills that we need to discuss how to improve our wellbeing and the wellbeing of others” [25]. Oades et al. claimed that positive education needs to further develop and implement wellbeing literacy to promote wellbeing [25]. As Oades et al. suggest, a common language should be a part of wellbeing literacy as the it affects thought, action, and ultimately outcomes [25].

Following, we present a framework for implementing sustainable wellbeing literacy within schools, using ten guiding rules (see Table 2). Given the sustainable wellbeing framework’s ultimate goal of changing various aspects of human behavior—regarding self-care and caring for the planet—an explicit, systematic educational approach to behavioral change should be applied. We propose that the cognitive-behavioral-therapeutic orientation may furnish a relevant theoretical foundation [76,77,78] and appropriate goal-directed tools [79,80] to support positive sustainability educational undertakings. The basic cognitive-behavioral model and its systematic tools for facilitating effective behavioral change have been successfully applied in various policy fields to create sustainable change [76,77,81]. Specifically, we recommend applying a set of ten rules based on Ronen’s [80] expansion of Kanfer and Schefft’s [79] six traditional thinking rules for therapists. The following sections develop and demonstrate these ten rules, using insights from both positive education and environmental education [33,82]. The proposed framework sets the grounds for implementing sustainable wellbeing literacy, guiding how to change thoughts, emotions, and behavior while striving for sustainable wellbeing. While our examples relate to specific topics and age groups, the implementation of the rules should fit any age group and may even be implemented with adults.

### 5.1. Focus on Behavior

Based on cognitive behavior theories, the first thinking rule suggests a more effective way to describe a real-life event in terms of the actions that caused it or the actions that may solve it rather than a static situation [79]. The goal of this first rule of our proposed “sustainable wellbeing literacy” would be to set a language that highlights the possibility of change and enables it, as situations seem fixed and unchangeable, whereas behaviors may be changed. It also implements the importance of the hope mechanism that was found to be imminent both in inducing environmental behavior and in raising subjective wellbeing [67,72,82,83,84,85]. The hope construct suggests that goals may be achieved following different paths [86]. The “think behavior” rule’s language enables thinking about behavioral paths to achieve a behavioral change.

Hence, “think behavior” helps define environmental situations in terms of the behaviors that created them or the actions that may solve them. For instance, instead of presenting overconsumption as an environmental problem, which highlights its situational components, the problem should be redefined in terms of its behavioral cause—in this case, the human action of buying too many products [62]. Importantly, especially for children, it is difficult to identify cause-effect linkages when the discussion of a troubling environmental situation is remote from its specific behavioral causes [87]. Furthermore, in light of one of the insights gleaned from research in the environmental education discipline, making the problem personal might be an effective method to “think behavior” [64,65]. Hence, redefining the problem of climate change in terms of students’ behavior should offer a much more effective approach to enhancing sustainable wellbeing literacy than discussing the disconnected major environmental problem or large-scale economic causes [64,65]. (See Table 2 for additional examples.)

From a positive psychology perspective, presenting our share in environmental problems and our behaviors’ changeability may help students feel that they can cope with the general sense of helplessness associated with major environmental problems, where people feel they lack any behavioral routes for impacting them [64,85]. Once an environmental issue is translated into human behaviors that should be performed, children may begin to believe that change is possible. Hence, their hope levels increase which in turn influences their individual wellbeing [15,67,73].

### 5.2. Focus on Solutions

Based on cognitive-behavior theories, the second principle of change draws our attention to the solutions instead of the causes of problems [79]. For instance, environmental programs may teach students about the world’s current linear industrial economic system, even tracing its roots back to the industrial revolution. This approach may provide theoretical and historical knowledge. However, if we want change to happen, the proposed positive sustainability approach would place greater emphasis on possible practical solutions to real current problems. Students should play an active role in generating effective solutions [62]. For example, in educating for sustainable consumption, students can be encouraged to think of practical solutions by asking: Where can we buy sustainable products? How can sustainable production be implemented? To provide role modeling and to stimulate brainstorming, whole-school programs can present best practices such as “cradle to cradle” concepts [88,89] as well as best performers who have adopted such pro-environmental solutions such as companies that lend clothes instead of selling them, or even a carpeting service: Instead of buying a carpet, the company replaces the old carpet and reuses it to manufacture a new one.

To help students “focus on solution,” many tools for establishing goals and pursuing them may be applied from the positive education field [48]. For instance, Mitra showed that working in groups to find solutions can promote children’s creativity and raise their curiosity and motivation [90]. Another example is the WOOP tool (wish, outcome, obstacle, plan), a simple, evidence-based technique that can enable students to think about possible solutions and expected obstacles to achieving them [91,92,93,94,95,96,97,98]. It was created to help people commit to a desired goal and set up paths toward achieving it, while specifically planning how to overcome potential obstacles. Hence, the “focus on solutions” rule implements another aspect of goal-oriented hope [86]: the pathway thinking, as it is directed to enhance the ability to find solutions to different problems.

Empirical research findings indicated that students’ goal-oriented environmental hope was connected to both sustainable wellbeing outcomes: to their subjective wellbeing and to their environmental literacy [67,73]. Both wellbeing outcomes where higher when school programs encouraged children to address solutions to environmental programs in their curriculum [67].

### 5.3. Be Flexible

Based on positive psychology, the Be Flexible rule involves finding multiple solutions to consider when approaching a problem, and multiple paths to overcome impending obstacles [79]. This positive sustainability principle is compatible with the pathway aspect of goal-oriented hope [87]. Flexibility is vital because pro-environmental change can be difficult to initiate, and new barriers can appear. Especially considering the enormously complex interrelations inherent to ecosystems and human systems, students’ adaptability is crucial. In line with a positive perspective, maintaining openness to various possible options can enable students to change their course of action once one path fails, without giving up on the end goal. Thus, the WOOP technique [92,93,94,95,96,97,98,99] could be useful for implementing this rule as it outlines plans for overcoming obstacles on the way to achieving desired outcomes.

As an example of the flexible rule, students’ goal may be to reduce air pollution by taking small steps to decrease emissions from driving a private car. Flexible thinking will enable students to set out multiple paths toward achieving that goal despite obstacles, such as walking by foot to closer destinations, cycling for longer distances, and using different forms of public transportation when it rains. Outlining different paths to achieve one’s goal induces innovation and creative ways of thinking, feeling, and behaving.

### 5.4. Think and Direct Behavior to the Future

Based on positive psychology—Think and Direct Behavior to the Future—focuses explicitly on forward-thinking instead of dwelling on the past [79]. This rule can be especially beneficial when confronting environmental issues, when young people may often feel angry and resentful about the environmental damage caused by previous generations. Positive psychology focuses on a shift in aim, from mitigating problems to the aim of promoting desired outcomes [99]. In this regard, instead of playing a “blame game” concerning past actions or dwelling on the upsetting current state of affairs in the natural world, students’ efforts and attention can be directed squarely toward delineating the specific future behavioral steps necessary for improving the state of the planet. The Future rule is also related to hope as it suggests focusing on a future goal and directing us for focusing on ways to solve it [15,67,73,86].

One example of implementing this rule in the educational context could be how teachers refer to endangered and extinct species. The focus on an elementary school discussing endangered species could take a more practical future-oriented focus while trying to think about solutions to protect current species in a specific habitat (preferably with hands-on implementation in a small garden) [67,73].

Thus, the combination of the four first rules: think behavior, focus on solutions, be flexible, and think and direct behavior towards the future offer a promising method for increasing students’ hope, that has been connected to both pro-environmental behavior and a higher level of sustainable wellbeing [15,67,73,85]. These rules guide the ways we approach and discuss environmental issues while developing and enhancing mechanisms that have been found to promote sustainable wellbeing.

### 5.5. Act in Small Steps

When people have major goals that seem very distant from their current situation, they may feel unable to begin taking any steps that can lead to change [79,100]. Alternatively, this principle advocates breaking up a formidable task like protecting the environment into small, achievable tasks. Hence, having already adopted the “solutions” principle, students can next divide their goals for large-scale environmental solutions into discrete viable steps, thus fostering a shift from inaction to action and enhancing motivation. An important aspect of the small steps rule is the notion of focusing on the near future and on possible immediate narrow actions, rather than on long-term and broader goals.

Connecting the “small steps” rule with the “ solution” rule, once again, the WOOP tool [91,92,93,94,95,96,97,98] offers a helpful option for effectively dividing the larger goal into discrete, measurable steps of gradually ascending difficulty while predicting the various kinds of obstacles that may arise. For instance, if students want to stop using plastics, they may start by bringing a personal water bottle to school, followed by using a non-disposable food container for lunch. Later, to extending the impact of their behavior change, they may plan a series of achievable advocacy activities targeting their family first, then their immediate friends, then their classroom, and finally, the school. Eventually, students can enlist others’ help in devising small steps to reach out to their communities and encourage neighbors, businesses, and organizations to join the effort to reduce their plastic usage.

### 5.6. Think and Feel Positive

Arguably, this rule captures the essence of sustainable wellbeing as it focuses on the convergence between pro-environmental behavior and individual wellbeing. Environmental behaviors might be perceived as taking their toll on individual happiness, thus creating barriers to exercising such behaviors [101,102]. The “think and feel positive” rule encourages finding the benefits of acting environmentally instead of focusing on their negative aspects, thus inducing positive emotions towards environmental behavior.

For instance, instead of thinking about what the students lose when reducing consumption of “fast fashion”, students can be encouraged to contemplate the various positive outcomes of their action: We will save money, save time, and have more resources for other activities that may have a more significant impact on our wellbeing. Likewise, we will potentially save planetary resources and protect weak populations whose laborers could be exploited and who could suffer from water pollution due to cheap toxic dyes. Focusing on these positive byproducts of planned pro-environmental behavioral changes can help motivate students to take action and help remind them of the rationales justifying their newly learned behaviors during times of doubt or frustration, such as when peer pressure elicits a desire for a new fashion purchase.

Similarly, students can reframe their loss due to the reduction of private car usage and emphasize the anticipated multiple positive benefits (such as enhancing our health by performing physical activity (walking, cycling) as alternatives to driving; enhancing our mindfulness to our surrounding environment and nature; connecting us to others we meet while walking, cycling, and riding buses; and, of course, reducing CO_2_ emissions to help contribute to decreasing global warming).

O’Brien’s “sustainable happiness list” could be useful in this regard, where students are encouraged to observe the impact of activities that increase their happiness on other members of society and the natural environment [16]. In other words, comprehensively examining the impacts of their own fulfilling new pro-environmental behaviors can help elucidate the interdependencies between personal wellbeing, societal wellbeing, and environmental wellbeing.

### 5.7. Identify and Use Strengths

Identify and Use Strengths–coincides with extensive research supporting the connection between using one’s character strengths and various aspects of wellbeing [48,103,104,105,106], especially within the school setting [48,107,108,109]. In the early 2000s, Peterson, Seligman, and colleagues identified 24 human character strengths that constitute the best of humans’ personalities [110]. Since the identification of character strengths, many studies have been performed to analyze the connections between character strengths and various aspects of wellbeing. Most of these studies are summarized in the main character strengths website (https://www.viacharacter.org/), where it is also possible to fill up a questionnaire to find out your signature strengths.

Each individual has a unique combination of strengths, and some of these strengths are called “signature strengths”: “strengths of character that a person owns, celebrates and frequently exercises” [110] (p. 18). A character strength would be considered a signature strength if it is a part of the person’s identity, the person frequently uses it, and using the strengths fills that person with energy [110]. Each person has three to eight signature strengths. Using signature strengths has been associated with many aspects of wellbeing [103,105,111,112,113].

In the positive education context, empirical evidence shows that character strengths can be enhanced through explicit teaching [48]. Teaching to identify and use character strengths in schools promotes students’ personal wellbeing [48,106,107,108] and, to some extent, their academic achievements [48,107,109]. It is also possible to develop specific character strengths such as curiosity, creativity, love of learning, and hope [48] and self-control skills [100,114,115].

In the environmental context, specific learning of character strengths such as appreciation of beauty, creativity, perspective, and self-regulation was found to be associated with people’s pro-environmental behavior [13]. In particular, zest, leadership, kindness, humility, prudence, fairness, and forgiveness were related to environmental self-efficacy [116], while curiosity has been linked to positive learning outcomes and, specifically, to the utilization of better learning strategies regarding environmental issues [117].

Thus, specific character strengths such as curiosity may be predicted to have the potential for improving both subjective wellbeing and environmental literacy, facilitating sustainable wellbeing. Therefore, one venue of the rule: “identify and use strengths” would be identifying specific strengths that are mostly connected with sustainable wellbeing and developing them.

The second venue of the rule would be encouraging the use of signature strengths. Students should ask themselves: How can each of our signature strengths be used to solve the specific problem? Identifying signature strengths may be a self-guided task, using the VIA questionnaire or can involve peer feedback; sometimes, students can effectively identify their peers’ strengths even while remaining less aware of their own.

For instance, if a school sets a goal of reducing its paper consumption, each student could use his signature strengths to achieve the desired goal. One student could use creativity to think about diverse ways to avoid the school’s traditional use of paper; another student could use social intelligence to win over the administrative staff and persuade them to stop printing their emails; a third student could rely on courage to confront a gruff teacher who printed an assignment on paper instead of preparing it on the school’s web service, and so on. This type of strength-based action can help empower students, enhancing their motivation and commitment to sustainability efforts.

### 5.8. Together and Integrative

Deriving from positive education tenets combined with the notion of “collaborative management” in public policy, “together and integrative” suggests that big changes almost always require that many people work collaboratively to achieve mutual goals [118]. Both logistically and psychologically, collective thinking and acting offer many benefits. When we work collaboratively, we can nurture one another, promote each other’s ideas, support one another when facing obstacles, and diversify our thinking because others from different backgrounds, professions, and roles can complement our knowledge and experience [118]. When we are not alone, the change we can achieve is much greater. Students in sustainable wellbeing programs should be encouraged to identify fruitful partners along the way, how they can best become involved, and how everyone can work together to bolster motivation and increase the effectiveness of their efforts.

Mitra suggested that children in groups attain the understanding that extends significantly beyond each individual in the group [90]. This collective “hive” mind works like an efficient teacher. Mitra’s model for innovative education underscored group project-based learning as crucial for efficient learning [90]. He emphasized the centrality of raising children’s curiosity and engagement by challenging them to collaborate—to think together—while exploring, asking questions, experiencing, and experimenting. These principles for innovation in education are easily relevant to positive sustainability education programs.

### 5.9. Find Resources

Based on positive education, the Resources rule accentuates the need to keep in mind the potential of internal and external supports and assets. When attempting to achieve the desired solution to a large-scale environmental problem, students will face many obstacles and challenges. A helpful way to prevent a sense of futility and despair is to consider multiple available resources supplied by other people and the natural environment [119,120]. For instance, resources could entail skills of schools’ stakeholders or other community members. To address a goal of water conservation, for example, students could be encouraged to think of rainwater as a natural resource that they can collect in special containers and use as gray water in toilets. If one of the parents works in construction, they could serve as a human resource to help engineer such a device.

### 5.10. Look at Policy and Policymakers

The final rule—Look at Policy and Policymakers—derives from policy considerations. For an effective change to occur both bottom up and top down approaches are required [121]. This rule encourages students to think about the following: When is the right time to involve policymakers in the process? How can local government, state, or country managers contribute to improving the state of the environment?

Stressing the need for education that emphasizes sustainable development, Grund and Brock recommended providing students with practical, real-life ways to influence policy [122]. They suggested activities such as discussing occupational choices and exploring ways in which students can impact the political domain as important components of effective environmental education. “Look at Policy and Policymakers” thus involves students in real-life processes, which coincides with positive education and the recommended 21st-century competencies [75]. Both parents and teachers can expose students to environmental policies and policymakers through media and literature and help students to meet and interview relevant environmental stakeholders in vivo. Involving students in real-world environmental activities at the political level also helps students balance their individual goals with collective ones by exposing them to the community, statewide, national, and international concerns, and perspectives. The broader real-world engagement with policymakers is also compatible with a comprehensive consideration of all wellbeing aspects—individual, societal, and environmental.

## 6. Conclusions

Today’s leaders of educational frameworks must prepare students to meet the recommended 21st-century skills and competencies (among which the four consensual ones are critical thinking, communication, collaboration, and creative problem solving) [75]. Considering the shared aspiration of positive education and environmental education disciplines to promote wellbeing, the proposed integrative conceptualization of the positive sustainability education framework can derive from best practices in the two separate fields (see Table 1).

Promoting wellbeing is an important objective at the national and international levels, encompassing the wellbeing of individuals, society, and the natural world. Since schools play a major role in designing humankind’s future, many educational programs have been created to promote wellbeing. However, while many advancements have evolved within separate educational strands, a holistic approach to the various aspects of wellbeing has been lacking, to create a common language, systems thinking, and integration of these separate theoretical and practical domains.

This paper reviewed how both positive education and environmental sustainability education approaches have promoted wellbeing. We proposed a mutual holistic definition of wellbeing, sustainable wellbeing, which includes the main components from both the positive psychology lens and the sustainability lens, acknowledging the interconnections between individuals’ wellbeing and the wellbeing of the natural environment (Figure 1).

Still, extant literature separately relates to wellbeing from the perspective of each lens. Hence, we proposed using a common language, educating students towards the concept of sustainable wellbeing. We propose that our sustainable wellbeing literacy model (See Table 2) can offer such a common language, to serve as a comprehensive theoretical framework for whole-school educational programs, which blends the positive psychology and environmental sustainability disciplines (see Table 1).

In order to produce a behavioral change, we proposed using sustainable wellbeing literacy, based on a cognitive-behavioral thinking approach combined with positive psychology, sustainability, and systems approach. We demonstrated how promoting sustainable wellbeing could benefit from deliberately planning the desired change.

Using the prevailing infrastructure of whole schools’ certification systems, we proposed including guidelines within the certification requirements to promote sustainable wellbeing (Table 1). Moreover, the theoretical framing of the various definitions of wellbeing could be mandatory conceptual components for whole-school programs, with the intent to elucidate the correlates between the individual, social, and natural world aspects.

The ten rules for implementing sustainable wellbeing literacy are proposed to help whole-school programs incorporate not only cognitive-behavioral principles but also components of innovation in education addressed for treating students as active participants, learning from experiences, and application. Thus, children will be asked to actively act as researchers (to raise their questions and hypotheses about topics related to sustainable wellbeing and search for relevant knowledge) rather than merely listening to teachers’ presentations of the research inquiry method. Positive education components of the proposed framework target positive relations between the student and peers, the student and teachers, and the student and parents that enable more in-depth learning while focusing on resources, strengths and building hope (Table 1). While some parts of our model were empirically tested, future research should design educational programs based on our proposal and empirically test their effectiveness in promoting sustainable wellbeing.

The current paper focused on the implications of the positive sustainability framework for education, but the framework itself is much broader and can be useful in many other areas. For instance, the framework could be used in various policy settings to achieve improved policy planning and results [123]. We hope that our elaborated program design will inspire both researchers and educators to explore further how a common language of sustainable wellbeing may be implemented and to achieve a future society that will better care for the planet and achieve higher levels of human wellbeing.

## Figures and Tables

**Table 1:** Implementation of main concepts in positive education, environmental education, and the integrated positive sustainability perspectives.

Concept	Positive Education Perspective	Environmental Education Perspective	Integrative: Positive Sustainability Education Perspective
Human wellbeing	The main focus (the main goal of positive education is to improve students’ individual wellbeing.)	Only as interconnected with the natural environment	A focus (in the integrated approach, the students’ individual wellbeing is one goal among others (such as protecting the natural environment).)
Natural environment	Not a main focus (positive psychology focuses on humans. The natural environment is only addressed (if at all) in connection to the human wellbeing.)	The main focus (protecting the natural environment and caring for it is the essence of environmental education.)	A focus (in the integrated approach both caring for the natural environment and individuals’ wellbeing will be at the left of the educational system.)
Teacher-Students relationship	A main focus (positive Education highlights the important role of teacher-students relationship.)	Not addressed	A main focus
Literacy	No prevailing concept (the concept of literacy is rarely discussed in positive education literature.)	Elaborated concept (environmental literacy is at the core of environmental education literature.)	Sustainable wellbeing literacy (the concept of sustainable wellbeing literacy is suggested to be at the core of the combined approach of positive sustainability education.)
Certification	None	Widespread international systems	Use the EE certification as a basis
Skills	A main focus (positive psychology focuses on imparting skills.)	Not a main focus (currently environmental education is less focused on the necessary skills students should acquire.)	A main focus
Teaching methods	E.g., relationship, Strengths focus	E.g., Outdoor learning, Engagement, experiencing	Combination
System thinking	No	A main focus	A main focus
Main discipline	Positive psychology	Environmental sciences	Multidisciplinary

**Table 2:** Main characteristics of the ten thinking rules, implementing sustainable wellbeing literacy, and examples of addressing the same topics with and without the rules.

Name	Goal	Example of Framing Environmental Issues	
		Without the Rule	Using the Rule
1. Focus on behavior	Highlights the possibility of change and enables it, focusing on actions rather than situations.	Overconsumption; Climate change: arctic melting, rising temperatures, flooding, extinct species, etc.	Buying too many products; buying stuff we do not need; switching off electrical devices not in use; using public transportation, walking, cycling.
2. Focus on solutions	Focusing on promoting desired outcomes, rather than on problems.	Industrial revolution causes and effects.	Where can we buy sustainable products? How can sustainable production be implemented? Students may generate solutions such as favoring second-hand clothing, continuing to wear the same garments for longer, or purchasing ethical fair-trade fashion brands.
3. Be flexible	Showing multiple options exist rather than one fixed solution.	Reducing private car usage.	Outlining different options for reducing car use (i.e., walking, cycling, public transportation).
4. Think and direct behavior to the future	Forward thinking instead of dwelling on the past.	Causes of extinct species.	How can we protect endangered species and their habitats?
5. Act in small steps	Breaking major goals into smaller gradual ones.	Stop using plastics; Protect endangered species.	First using reusable bottle; following—other personal actions; following—effecting family; following—advocating policy. First add nesting places for birds; following—plant flowers for bees; following—request no herbicides in a local garden; following—advocate no herbicides in the city; following—advocating legal ban of herbicides within urban parks.
6. Think and feel positive	Thinking about the positive outcomes of environmental behavior, the convergence between individual and subjective wellbeing rather than the negative consequences of the behavior.	My happiness is reduced when I do not buy enough fast fashion clothes or when I reduce my private car use; I lose time and convenience.	We will save money, save time, and have more resources for other activities that may have a more significant impact on our wellbeing, help others and the environment; Acknowledge multiple benefits: environmental, other species, health, connections with others
7. Identify and use individual strengths	Using signature strengths to address environmental issues and develop specific related strengths rather than following one standard route.	Reducing paper consumption in school using a single plan.	Each student uses his own signature strength to address the issue: social intelligence—persuade administrative staff; creativity—suggest different solutions; courage—confront a teacher.
8. Together and integrative	Working together with others to achieve the goal facilitates the change process.	I will do my best alone.	Who are the people around that could be the partners for action.
9. Find resources	Use all resources for solving a problem.	We do not have the budget to build graywater system at school.	One of the parents is an engineer who can build such a system.
10. Look at policy and policymakers	Scaling up wellbeing.	My role ends with my behavior.	Scaling up the problem to the policy level.
